# Opposing Molecular Programs in Obsessive-Compulsive Disorder and Hoarding: Transcriptome-Wide Association Studies Reveal Distinct Senescence, Complement, and Metabolic Signatures

**DOI:** 10.7759/cureus.110702

**Published:** 2026-06-11

**Authors:** Ngo Cheung

**Affiliations:** 1 Psychiatry, Cheung Ngo Medical Limited, Hong Kong, HKG

**Keywords:** ageing, complement, hoarding disorder, metabolic, mitochondrial senescence, ocd/ anxiety disorders, twas

## Abstract

Background and objective: Obsessive-compulsive disorder (OCD) and hoarding disorder often co-occur but differ in onset, course, symptoms, and treatment response. This study compared diagnosis-based OCD genetic liability with hoarding-symptom liability, rather than direct expression differences between diagnosed patient groups.

Methods: Large-scale OCD and hoarding genome-wide association study (GWAS) summary statistics were integrated with brain expression quantitative trait loci (eQTL) reference panels using a transcriptome-wide association approach. Gene-set enrichment was assessed across six brain regions with Stouffer’s Z-score meta-analysis, empirical permutation testing, paired phenotype comparisons, and exploratory gene-level analyses. Transcriptome-wide association studies (TWAS) Z-scores were interpreted as genetically predicted expression associated with trait liability, not measured tissue expression.

Results: The OCD liability showed positive intrinsic apoptosis enrichment (Z = +5.707; permutation p = 0.005899), positive complement enrichment (Z = +4.932; p = 0.0164), and a negative synapse-pruning profile (Z = −4.802; p = 0.0178). Hoarding-symptom liability showed a more positive cellular-senescence profile than OCD (Mann-Whitney U p = 0.008861; local false discovery rate (FDR) = 0.008861). Nicotinamide adenine dinucleotide (NAD)/sirtuin (SIRT) findings were directionally hoarding-skewed but exploratory.

Conclusions: These findings suggest divergent, hypothesis-generating molecular signatures, namely senescence/NAD-biased aging signals in hoarding and apoptosis, complement-pruning, and metabolic-reward dysregulation in OCD. The TWAS findings require validation with colocalization, fine-mapping, and functional studies.

## Introduction

Clinical and genetic context

Obsessive-compulsive disorder (OCD) and hoarding disorder are closely related conditions, but they are not interchangeable. The former is defined by intrusive thoughts and repetitive behaviors, typically beginning in late childhood or early adulthood, and has a lifetime prevalence of about 2% to 3% [1]. The latter disorder is marked by persistent difficulty discarding possessions, excessive accumulation, and the resulting clutter and impairment [1]. Its prevalence in the general population has been estimated at 2% to 6%, and symptoms often become more evident in middle and later life [2,3]. Although hoarding may occur with OCD, hoarding disorder does not require the intrusive obsessions and repetitive compulsions that define OCD [1,4]. This difference in core psychopathological manifestation is central to the distinction between the two conditions. Although many people with hoarding symptoms also meet criteria for OCD, many do not, and hoarding symptoms often respond less well than other OCD dimensions to first-line serotonin reuptake inhibitor treatment [4-6]. These differences have sustained the question of whether hoarding is best understood as a subtype of OCD or as a related but partly distinct condition [4].

Obsessive-compulsive symptoms (OCS) refer to dimensional intrusive thoughts, urges, or repetitive behaviors that may occur across a range of severity, including below the threshold for a formal OCD diagnosis. In contrast, OCD refers to a clinical diagnosis based on symptom pattern, distress, impairment, and duration [1]. Because the primary obsessive-compulsive input in the present analysis was a diagnosis-based OCD genome-wide association study (GWAS) rather than a dimensional OCS GWAS, the results below use OCD liability for the transcriptome-wide association studies (TWAS) findings. The OCS is used only when discussing dimensional symptoms more generally. Likewise, the hoarding input was a hoarding-symptom GWAS, so the analytic results are described as hoarding-symptom liability rather than confirmed hoarding disorder [7].

Genetic findings support both overlap and separation. Twin studies place OCD heritability at roughly 40% to 50% [8], while hoarding symptoms show heritability estimates ranging from 26% to 48% across cohorts [7]. Twin-based heritability estimates the proportion of trait variation attributable to genetic influences inferred from resemblance between relatives, whereas single-nucleotide polymorphism (SNP)-based heritability estimates the proportion of variance explained by commonly measured or imputed SNPs captured in GWAS data. The SNP-based estimates are usually lower because they do not capture all genetic variation, rare variants, or every source of inherited liability. A meta-analysis of hoarding symptoms in more than 27,000 individuals did not identify genome-wide significant loci, although SNP-based heritability was estimated at 11% [7]. By contrast, a 2025 OCD GWAS meta-analysis that included 53,660 cases and more than two million controls identified 30 independent genome-wide significant loci and implicated about 25 credible genes, many expressed in cortico-striato-thalamo-cortical (CSTC) circuitry [9]. Genetic correlation analyses have generally pointed to positive but incomplete overlap between OCD and hoarding symptoms, suggesting that shared liability coexists with meaningful disorder-specific components [7]. Even so, the transcriptomic mechanisms that separate the two phenotypes remain poorly defined.

Rationale for molecular investigation

Clinical and epidemiological patterns suggest that OCD and hoarding may diverge biologically in systematic ways. Hoarding often shows later onset or progressive worsening and is associated with executive dysfunction, impaired decision-making, and age-related cognitive change [2,10]. Those features raise the possibility that hoarding is linked to cellular aging processes in prefrontal and striatal networks. Obsessive-compulsive disorder, in contrast, more often begins earlier and has repeatedly been connected to neurodevelopmental abnormalities, including altered synaptic pruning and immune signaling within CSTC loops [11-13]. Patient-level studies have also reported markers consistent with accelerated cellular aging in OCD, including shorter telomeres and reduced mitochondrial DNA copy number [14]. Together, these findings suggest that the two phenotypes may reflect partly opposing molecular programs: one weighted toward senescence and metabolic aging and the other toward immune and developmental dysregulation.

Transcriptome-wide association studies provide a practical way to test that idea. By integrating GWAS summary statistics with expression quantitative trait locus data, TWAS can identify genes whose genetically predicted expression is associated with disease risk [15]. An expression quantitative trait locus (eQTL) is a genetic variant associated with variation in gene expression. In TWAS, eQTL weights are used to predict the genetically regulated component of expression and then test whether that predicted expression is associated with a trait [15]. In psychiatric genetics, this approach has been useful for moving beyond GWAS loci alone and toward candidate genes and pathways that are easier to interpret biologically. A comparative TWAS framework, therefore, offers a way to move from broad genetic overlap to more specific hypotheses about what OCD and hoarding share and where they differ.

Focus on specific pathways

Several interconnected systems were prioritized because they map directly onto the clinical hypotheses. Nicotinamide adenine dinucleotide (NAD) is a central metabolic cofactor involved in redox reactions and cellular energy balance. The NAD+ refers specifically to the oxidized form of NAD, which accepts electrons in metabolic reactions and also serves as a substrate for enzymes such as sirtuins (SIRT) and poly(adenosine diphosphate (ADP)-ribose) polymerases (PARP) [16,17]. The NAD metabolism and SIRT signaling were included because NAD+ is a core cofactor for SIRTs, which influence aging, mitochondrial function, DNA repair, and inflammatory regulation [16]. Sirtuins are NAD+-dependent deacylase/deacetylase enzymes that regulate chromatin state, mitochondrial function, stress responses, DNA repair, and metabolism [16]. Disruption of this axis has been implicated in brain aging and neurodegeneration [17].

Cellular senescence pathways were included to test the aging-related hypothesis for hoarding more directly. Cellular senescence is a durable stress-response state in which cells stop dividing and often adopt a proinflammatory secretory phenotype. It is closely linked to aging because senescent cells can accumulate over time and contribute to chronic inflammation, tissue dysfunction, altered mitochondrial signaling, and impaired repair capacity [18]. Senescence involves durable cell-cycle arrest together with a proinflammatory secretory phenotype, mitochondrial dysfunction, and stress signaling, all of which could contribute to rigidity in neural circuits and executive impairment [18].

Glucagon-like peptide-1 (GLP-1) and insulin secretion pathways were examined because incretin signaling has growing relevance to metabolic regulation and may intersect with reward-related behavior. The GLP-1 is an incretin hormone released mainly from intestinal L cells and certain brainstem neurons [19]. Incretin signaling refers to gut-derived hormonal signaling that enhances glucose-dependent insulin secretion after nutrient intake. Insulin secretion pathways, therefore, overlap with GLP-1 biology because GLP-1 receptor activation can amplify pancreatic insulin release and influence central circuits involved in satiety and reward [19-22]. Synaptic pruning, complement activation, and glutamatergic plasticity were also prioritized. Synaptic pruning is the selective elimination of synapses during development and experience-dependent remodeling. Complement activation refers to engagement of innate immune proteins, including C3 and C4 family members, that can tag synapses for removal [23]. Glutamatergic plasticity refers to activity-dependent strengthening or weakening of excitatory synapses [11,12]. Complement proteins mark synapses for microglial elimination during development, and altered pruning has been proposed as one route to compulsive pathology [11,13,23].

Finally, apoptosis and mitochondrial pathways were included as candidate shared stress-response systems. Apoptosis is programmed cell death regulated by intrinsic mitochondrial signals and extrinsic receptor-mediated signals. Mitochondrial pathways include oxidative phosphorylation, mitochondrial dynamics, mitochondrial DNA maintenance, and mitochondrial control of apoptotic thresholds. These pathways may operate across both conditions in the setting of altered cortico-striatal circuit function [13,17]. These systems converge on circuits implicated in both disorders and include several druggable nodes, such as NAD precursors, senescence-modifying strategies, GLP-1 receptor agonists, and complement-related interventions [16,17,19,22,24,25].

## Materials and methods

Study design and ethical considerations

This was an in silico computational post-GWAS integrative study based on publicly available or collaborator-provided summary statistics. It combined GWAS, TWAS, and curated pathway enrichment analyses. No new participant recruitment, intervention, or individual-level clinical data analysis was performed. As the study used de-identified summary-level genetic data, it did not involve direct human-subjects procedures; therefore, this study did not require Institutional Review Board (IRB) approval.

GWAS input data

The analysis used summary statistics from two large GWAS meta-analyses. The GWAS on hoarding symptoms combined seven international cohorts and included 27,537 individuals of predominantly European ancestry. Hoarding phenotypes were based on parent- or self-report. No genome-wide significant loci were identified, but SNP-based heritability in an unrelated subsample was estimated at about 11% [7]. The OCD GWAS included 28 cohorts and comprised 53,660 cases and 2,044,417 controls, with an effective sample size of approximately 210,000. This study identified 30 independent genome-wide significant loci and implicated about 25 credible genes [9]. Both datasets were drawn from publicly available sources or direct collaboration with the original study teams.

The difference in statistical power between the two GWAS was considered during interpretation. The OCD GWAS was substantially larger and yielded genome-wide significant loci, whereas the hoarding-symptom GWAS was smaller and did not identify genome-wide significant loci. This imbalance can increase TWAS detection sensitivity for OCD and can also make apparent sign differences harder to interpret, particularly for genes with weak or noisy hoarding associations. For this reason, cross-phenotype contrasts were interpreted as exploratory unless supported by consistent pathway direction, empirical testing, and tissue recurrence.

TWAS pipeline

Transcriptome-wide association analyses were performed using an S-PrediXcan-style framework with precomputed Genotype-Tissue Expression (GTEx) version 8 (v8) multivariate adaptive shrinkage (MASHR) expression prediction models (GTEx Portal, Broad Institute of MIT and Harvard, Cambridge, MA, USA). The models estimate the genetically regulated component of gene expression from GWAS summary statistics and do not measure expression directly [15]. Six GTEx v8 brain tissues were analyzed: amygdala, anterior cingulate cortex BA24, caudate basal ganglia, frontal cortex BA9, hippocampus, and nucleus accumbens basal ganglia.

The GWAS summary statistics were parsed and harmonized into an S-PrediXcan-compatible format. Variants were matched to model variants using panel variant identifiers where chromosome, position, and alleles were available, with rsID-based fallback matching when necessary. Alleles were aligned against the prediction-model effect and reference alleles, and Z-scores were sign-flipped when the GWAS effect allele and non-effect allele were reversed relative to the model.

Gene-level association Z-scores were computed for each tissue by summing SNP-level GWAS Z-scores weighted by the expression prediction weights and normalizing by the model covariance matrix. Genes with no matched SNPs between the GWAS summary statistics and the prediction model were excluded. No additional minimum SNP-count threshold was imposed. The analysis produced per-tissue S-PrediXcan association results, including gene-level Z-scores, p-values, number of SNPs used, number of SNPs in the model, and prediction-performance metrics when available from the model database. Tissue-specific results were concatenated into a combined results table across the analyzed brain tissues. No formal cross-tissue meta-analysis was performed in this pipeline.

Gene-set selection and rationale

A curated panel of 40 gene sets was assembled around four main themes. Gene sets were drawn from static curated gene-set files using standard pathway identifiers from Gene Ontology Biological Process, Reactome, KEGG, WikiPathways (WP), Hallmark, and related curated collections. The exact gene membership used in the run should be treated as defined by the archived gene-set files, because pathway database membership can change across releases.

The first cluster focused on NAD metabolism and SIRT biology and included WP NAD METABOLISM ALL, Kyoto Encyclopedia of Genes and Genomes (KEGG) NICOTINATE AND NICOTINAMIDE METABOLISM ALL, REACTOME NICOTINATE METABOLISM ALL, WP NAD BIOSYNTHETIC PATHWAYS ALL, and WP NAD METABOLISM SIRTUINS AND AGING ALL. These sets were chosen to test whether one phenotype showed stronger alignment with metabolic aging programs [16,17].

The second cluster covered cellular senescence and included GOBP CELLULAR SENESCENCE ALL, Gene Ontology biological process (GOBP) REGULATION OF CELLULAR SENESCENCE ALL, REACTOME CELLULAR SENESCENCE ALL, REACTOME SENESCENCE ALL, SAUL SEN MAYO ALL, and REACTOME TELOMERE ALL. These sets directly addressed the hypothesis that hoarding may be more strongly linked to aging-related molecular programs [18].

The third cluster targeted GLP-1 and insulin biology. It included GOBP INSULIN SECRETION INVOLVED IN CELLULAR RESPONSE TO GLUCOSE STIMULUS ALL, REACTOME REGULATION OF INSULIN SECRETION ALL, WP GLP1 FROM INTESTINE AND PANCREAS AND ROLE IN GLUCOSE HOMEOSTASIS ALL, and related Reactome pathways. These were selected because incretin signaling may intersect with compulsivity, reward processing, and metabolic regulation [19-22].

The fourth cluster centered on synaptic pruning, plasticity, and immune processes. Included sets were GOBP SYNAPSE PRUNING ALL, REACTOME COMPLEMENT ALL, GOBP REGULATION OF SYNAPTIC PLASTICITY ALL, KEGG LTP ALL, and GOBP GLUTAMATERGIC ALL. Complement-mediated synapse elimination has particular relevance to neurodevelopmental and compulsivity models [11,13,23].

To place those findings in a wider biological context, the analysis also included supporting sets for mitochondrial and energy metabolism, such as MOOTHA MITOCHONDRIA ALL, HALLMARK OXIDATIVE PHOSPHORYLATION ALL, and MOOTHA PGC ALL; apoptosis pathways, including GOBP INTRINSIC APOPTOTIC SIGNALING PATHWAY ALL, REACTOME INTRINSIC PATHWAY FOR APOPTOSIS ALL, and HALLMARK APOPTOSIS ALL; cell-type signatures, including ADULT ASTRO ALL, ADULT OLIGO ALL, and ADULT MICRO ALL; and immune-related sets such as HLA COMPLEX ALL. Monoamine and cAMP-related sets were also retained as reference pathways relevant to established OCD models [5,6]. In total, the prespecified panel contained five NAD/SIRT sets, six senescence/telomere sets, four GLP-1/insulin sets, five synaptic-pruning/plasticity/immune sets, and 20 supporting apoptosis, mitochondrial, cell-type, immune, monoamine, and cyclic adenosine monophosphate (cAMP)-related sets.

Statistical analysis

Pathway enrichment was evaluated with Stouffer’s Z-score method and permutation-based p-values using 10,000 permutations. For gene-set enrichment, permutations were gene-label based: for each pathway, random gene sets of the same size were sampled from the analysis background, and the Stouffer statistic was recalculated to generate an empirical null distribution. This controlled for gene-set size but did not fully preserve local linkage disequilibrium (LD), correlated eQTL weights, or gene-gene dependence within pathways. Therefore, permutation p-values are interpreted as empirical gene-set-size controls rather than fully LD-aware competitive gene-set tests.

Mean Z-scores were computed separately for OCD and hoarding for each gene set. Paired t-tests and Wilcoxon signed-rank tests were then used to compare the two phenotypes directly. These paired tests treated the gene-level Z-scores within each gene set as paired observations, meaning that each gene’s Z-score under the OCD GWAS was directly paired with its Z-score under the hoarding-symptom GWAS. This approach tests whether the average within-gene difference in Z-scores between the two phenotypes differs systematically from zero. Because TWAS Z-scores are correlated through LD, shared cis-eQTL weights, and overlapping gene sets, these paired tests should be interpreted as exploratory profile-comparison tests rather than definitive independent-gene tests.

Meta-analysis across the six brain regions also used Stouffer’s method. At the gene level, candidate opposing genes were defined as genes with opposite directions of effect and large absolute differences in Z-scores. For pathway-level summaries, 'opposing' referred to phenotype-level mean Z-scores with opposite signs or clearly separated directions. Genes were selected when the absolute difference between OCD and hoarding Z-scores exceeded 2; 'strongly opposite' was reserved for opposite-sign gene effects with absolute Z-score differences greater than 5. Sign concordance rates were calculated to assess directional consistency. False discovery rate (FDR) correction was applied within local families of pathway contrasts using the Benjamini-Hochberg procedure. Because a single global or family-wise FDR correction across all pathways, tissues, cell types, and gene-level analyses was not available, findings are reported as permutation-supported, local FDR-supported, nominal, or exploratory, as appropriate.

All statistical analyses were performed using Python (Python Software Foundation, Wilmington, DE, USA). The exact Python version was not inferable from the exported pathway tables and should be reported with the final reproducibility archive. The cut-off for nominal statistical significance was set at p < 0.05. False discovery rate correction was applied using the Benjamini-Hochberg procedure within local thematic or differential testing families, with significance defined as FDR < 0.05.

Interpretation framework

In this study, opposing regulation referred to statistically meaningful differences in mean Z-scores between OCD and hoarding when the directions of effect were opposite. Concordant regulation referred to similar directional effects across the two phenotypes. A positive TWAS Z-score was interpreted as higher genetically predicted expression being associated with higher trait liability. A negative TWAS Z-score was interpreted as lower genetically predicted expression being associated with higher trait liability, or equivalently, an inverse predicted-expression association. These terms do not imply observed biological upregulation or downregulation in patient brain tissue.

The caudate and frontal cortex BA9 were prioritized in interpretation because of their central role in CSTC circuitry and because they produced relatively consistent signals across analyses [13]. However, findings in the nucleus accumbens, hippocampus, amygdala, and anterior cingulate cortex were also considered when pathway signals recurred across regions. Cell-type patterns were examined to help distinguish glial from neuronal contributions, especially in complement- and pruning-related pathways.

Sensitivity and robustness checks

Robustness was evaluated by examining whether pathway signals were supported by more than one statistic, including Stouffer Z-scores, empirical permutation p-values, Wilcoxon tests, recurrence across brain-region models, and leave-one-gene-out influence patterns. Findings dominated by very small gene sets or single genes were treated as lower confidence. No colocalization, eQTL-GWAS fine-mapping, or LD-aware competitive gene-set test was performed in the present analysis; therefore, gene-level candidates are not interpreted as proven causal genes [26,27].

Data and code availability

The GWAS summary statistics are available through the cited source studies and their access conditions [7,9]. Curated gene-set lists, intermediate TWAS outputs, pathway-level summaries, and analysis scripts can be made available from the author on reasonable request, subject to the data-use terms of the original GWAS and eQTL resources.

## Results

Overview of gene-set testing

Across the 40 gene sets, the comparison revealed a mix of shared and divergent signals between OCD liability and hoarding-symptom liability (see Appendix A for comprehensive results). The clearest contrasts emerged in pathways related to cellular senescence, complement signaling, synapse pruning, and NAD metabolism. Other pathways, especially apoptosis-related pathways, showed overlap across the two phenotypes, whereas broad mitochondrial signatures were comparatively weak. Overall, the data supported the presence of partially opposed transcriptomic profiles rather than a simple difference in magnitude along a single shared axis. Because many results were nominal or locally corrected rather than globally corrected, the findings should be read as discovery-stage and hypothesis-generating.

Strongest opposing pathways

The most biologically coherent divergence involved cellular senescence (Table 1). This pathway was considered one of the strongest divergences, not because it had the single largest mean difference in every test, but because it showed convergent evidence across direction, tissue recurrence, direct OCD-hoarding comparison, and biological fit with the clinical course of hoarding. The gene set GOBP CELLULAR SENESCENCE showed a negative OCD profile and a positive hoarding profile. In the direct differential analysis, hoarding showed a significantly more positive senescence profile than OCD, with a mean Z-score difference of approximately −0.872 for OCD minus hoarding, Mann-Whitney U p = 0.008861, local FDR = 0.008861, and paired Wilcoxon p = 0.04091. Meta-analysis across tissues reinforced the direction: hoarding showed a positive Stouffer Z of +3.331, while OCD showed a negative Stouffer Z of −4.296. Similar directional patterns were seen in GOBP REGULATION OF CELLULAR SENESCENCE and related senescence sets.

**Table 1:** Pathway-level opposite regulation between OCS and hoarding (paired analysis) Positive TWAS Z-scores indicate higher genetically predicted expression associated with higher trait liability; negative Z-scores indicate inverse predicted-expression association. Local FDR values refer to the available differential testing family and should not be interpreted as global family-wise correction across all exploratory analyses. REACTOME and WP indicate Reactome and WikiPathways gene-set sources. Mean difference = OCD minus hoarding OCS: Obsessive-compulsive symptoms, TWAS: Transcriptome-wide association studies, FDR: False discovery rate, OCD: Obsessive-compulsive disorder; GWAS:  Genome-wide association study; GOBP: Gene Ontology biological process; KEGG: Kyoto Encyclopedia of Genes and Genomes; NAD: Nicotinamide adenine dinucleotide, WP: WikiPathways

Gene set	Direction of difference	Mean difference (OCD − hoarding)	Main differential p-value	Local FDR	Secondary statistic	Interpretation
GOBP_CELLULAR_SENESCENCE_ALL	OCD lower than hoarding	−0.872	Mann-Whitney U p = 0.008861	0.008861	Paired Wilcoxon p = 0.04091	Hoarding shows a more positive genetically predicted cellular-senescence profile
REACTOME_COMPLEMENT_ALL	OCD higher than hoarding	+1.059	Mann-Whitney U p = 0.0368	0.0368	Paired Wilcoxon p = 0.00934	OCD shows a stronger complement-related predicted-expression profile
GOBP_SYNAPSE_PRUNING_ALL	OCD lower than hoarding	−1.176	Mann-Whitney U p = 0.03809	0.03809	Paired Cohen’s d = −0.56	OCD shows a more negative synapse-pruning profile
KEGG_NICOTINATE_AND_NICOTINAMIDE_METABOLISM_ALL	OCD lower than hoarding	−0.931	Paired Wilcoxon p = 0.03012	Not locally FDR-supported in the available summary	Paired Cohen’s d = −0.53	Exploratory hoarding-skewed nicotinate/nicotinamide signal
WP_NAD_METABOLISM_SIRTUINS_AND_AGING_ALL	OCD lower than hoarding	−1.066	p = 0.249	Not significant	Wilcoxon p = 0.275	Directionally opposite but small-set and exploratory

The complement pathway also clearly separated the phenotypes. The REACTOME COMPLEMENT showed a more positive OCD profile than hoarding, with a mean difference of approximately +1.059 in the differential analysis, Mann-Whitney U p = 0.0368, local FDR = 0.0368, and paired Wilcoxon p = 0.00934. The OCD complement signal was also supported by cross-tissue pathway enrichment, with Stouffer Z = +4.932 and permutation p = 0.0164. The dedicated synapse pruning set showed the reverse direction. The GOBP SYNAPSE PRUNING had a more negative OCD profile than hoarding, with a mean difference of approximately −1.176 and Mann-Whitney U p = 0.03809, and local FDR = 0.03809. The OCD synapse-pruning pathway was also negative in the cross-tissue enrichment analysis, with Stouffer Z = −4.802 and permutation p = 0.0178. The complement-pruning relationship, therefore, did not reduce to a single simple immune effect.

The NAD metabolism and SIRT-related pathways also showed notable opposition, although the evidence was more exploratory. The WP NAD METABOLISM SIRTUINS AND AGING had mean Z-scores that trended lower in OCD and higher in hoarding. The KEGG NICOTINATE AND NICOTINAMIDE METABOLISM showed a mean Z-score difference of approximately −0.931, with paired Wilcoxon p = 0.03012. Meta-analytic results suggested a positive hoarding signal in nicotinate metabolism and related NAD pathways, but these sets were often small and did not provide the same level of support as the senescence, complement, and synapse-pruning contrasts.

Concordant or weakly opposite pathways

Not every pathway showed a clear split (Table 2). Intrinsic apoptosis was upregulated in both phenotypes, although the effect was numerically stronger in OCS. REACTOME INTRINSIC PATHWAY FOR APOPTOSIS had mean Z-scores of +0.832 in OCS and +0.258 in hoarding, with a mean difference of +0.574. Meta-analysis showed a particularly strong OCS signal, with Stouffer Z = +5.707 and permutation p = 0.004. HALLMARK APOPTOSIS showed the same general pattern, although more weakly.

**Table 2:** Pathways with concordant or weakly opposite regulation Mean Z = Average gene-level TWAS Z-score across genes in the set; Mean difference = OCD minus hoarding; Sign concordance = proportion of genes in the set with Z-scores of the same sign in both OCD and hoarding analyses. Positive and negative values refer to genetically predicted expression associations with trait liability, not measured expression changes in patients. OCD: Obsessive-compulsive disorder; OCS: Obsessive-compulsive symptoms, TWAS: Transcriptome-wide association studies

Gene set	Mean Z_OCS_	Mean Z_Hoarding_	Mean difference	Paired t P	Sign concordance	Interpretation
REACTOME_INTRINSIC_PATHWAY_FOR_APOPTOSIS	+0.832	+0.258	+0.574	0.158	56.5%	Apoptosis activated in both; stronger in OCS
HALLMARK_APOPTOSIS	+0.175	+0.023	+0.152	0.243	58.7%	Mild shared pro-apoptotic signal
MOOTHA_MITOCHONDRIA	+0.112	−0.010	+0.123	0.412	51.0%	Near-neutral mitochondrial signal
HALLMARK_OXIDATIVE_PHOSPHORYLATION	+0.046	−0.062	+0.114	0.587	54.7%	Weak/neutral

By contrast, mitochondrial and oxidative phosphorylation pathways were largely neutral at the pathway level. MOOTHA MITOCHONDRIA and HALLMARK OXIDATIVE PHOSPHORYLATION showed small mean differences, non-significant paired tests, and sign concordance rates around 50% to 55%. This suggests that broad mitochondrial bioenergetics did not provide the main distinction between OCS and hoarding in this analysis.

Gene-level drivers of key signals

Several individual genes appeared to drive the strongest pathway-level differences (Table 3). Within the senescence and NAD-related sets, SIRT2 showed a Z-score of +2.61 in OCS and −3.14 in hoarding, giving a Z difference of +5.75 (Table 4). The nicotinamide phosphoribosyltransferase (NAMPT) showed a Z-score of −3.88 in OCS and +0.70 in hoarding, for a Z difference of −4.59. SIRT3 and SIRT4 also showed opposite directions between the two phenotypes. The 5′-nucleotidase ecto (NT5E) was upregulated in both conditions, but more strongly in hoarding.

**Table 3:** Top candidate genes contributing to differences in senescence, mitochondrial, and NAD/nicotinamide pathways Genes were selected by an absolute Z-score difference greater than 2. These are candidate contributors to pathway patterns, not confirmed causal genes. CAT: Catalase; MTCH2: Mitochondrial carrier 2; GLS2: Glutaminase 2; SIRT: Sirtuin; NAMPT: Nicotinamide phosphoribosyltransferase; NT5E: 5′-nucleotidase ecto; PARP10: Poly(adenosine diphosphate (ADP)-ribose) polymerase family member 10; NAD: Nicotinamide adenine dinucleotide; OCD: Obsessive-compulsive disorder

Gene	Pathway	Z_OCS_	Z_Hoarding_	Z difference (OCS − hoarding)	Interpretation
CAT	Mitochondria	+7.72	−0.81	+8.53	Strongly up in OCS
MTCH2	Mitochondria	−6.33	+2.02	−8.35	Opposite mitochondrial transport
GLS2	Mitochondria	−7.33	−0.50	−6.83	Strongly down in OCS
CD40	Mitochondria	−9.47	−3.24	−6.23	Strongly down in OCS
SIRT2	NAD / Senescence	+2.61	−3.14	+5.75	Up in OCS, down in hoarding
NAMPT	NAD metabolism	−3.88	+0.70	−4.59	Key NAD biosynthesis enzyme; opposite
NT5E	NAD metabolism	+1.34	+3.42	−2.08	Concordant up; stronger in hoarding
PARP10	NAD metabolism	−3.32	−1.30	−2.02	PARP family (NAD consumer)

**Table 4:** NAD metabolism gene-level detail of the SIRT family and related enzymes The Z-scores represent meta-analytic TWAS association strength across brain tissues. Positive Z = higher genetically predicted expression associated with higher trait liability; negative Z = inverse predicted-expression association; 'Strongly opposite' = opposite signs and absolute Z-score difference greater than 5; 'Opposite' = opposite signs and absolute Z-score difference greater than 2; 'Concordant positive' = both positive SIRT: Sirtuin; NAMPT: Nicotinamide phosphoribosyltransferase; NMNAT2: Nicotinamide nucleotide adenylyltransferase 2; NT5E: 5′-nucleotidase ecto; PARP1: Poly(adenosine diphosphate (ADP)-ribose) polymerase 1; NAD: Nicotinamide adenine dinucleotide; OCD: Obsessive-compulsive disorder, TWAS: Transcriptome-wide association studies

Gene	Z_OCS_	Z_Hoarding_	Direction summary
SIRT2	+2.61	−3.14	Strongly opposite
SIRT3	+1.51	−2.55	Opposite
SIRT4	−0.16	+3.30	Opposite (up in hoarding)
SIRT6	+1.30	+2.10	Concordant up
NAMPT	−3.88	+0.70	Opposite (down in OCS)
NMNAT2	+2.47	+1.86	Concordant up
NT5E	+1.34	+3.42	Concordant up (stronger in hoarding)
PARP1	−2.11	+1.43	Opposite

Within mitochondrial and related pathways, catalase (CAT) showed one of the largest opposite-direction effects, with +7.72 in OCS and −0.81 in hoarding, for a Z difference of +8.53. The mitochondrial carrier 2 (MTCH2) showed −6.33 in OCS and +2.02 in hoarding. The glutaminase 2 (GLS20) and CD40 contributed strongly, both downregulated in OCS (more markedly than in hoarding). These gene-level findings were consistent with the broader pathway results. These genes are best interpreted as candidate contributors rather than confirmed causal drivers. No colocalization or TWAS fine-mapping was performed in the present analysis, so gene-level associations may reflect linkage disequilibrium between GWAS and eQTL signals or correlation among prediction models.

Tissue and cell-type patterns

The strongest and most consistent signals were observed across cortico-striato-limbic regions. For OCD, intrinsic apoptosis was most prominent in the nucleus accumbens and hippocampus, while complement and pruning-related signals were evident across hippocampus, caudate, nucleus accumbens, frontal cortex, and anterior cingulate models. For hoarding, cellular senescence and the regulation of cellular senescence were most evident in the caudate, hippocampus, amygdala, and anterior cingulate models. This regional pattern is notable given the long-standing relevance of these regions to cortico-striato-thalamo-cortical models of compulsivity [10]. Meta-analytic Z-scores were generally more stable and more interpretable than single-tissue results, suggesting that the shared cross-tissue component captured the central disease-relevant signal.

Cell-type analyses suggested modest glial involvement. Adult microglial gene profiles showed one of the stronger cross-phenotype similarities, with OCD and hoarding profiles positively correlated across adult microglial genes (Pearson r = 0.636, p = 0.002575; Spearman rho = 0.603, p = 0.004887). Adult astrocyte profiles were also modestly similar (Pearson r = 0.373, p = 0.03551; Spearman rho = 0.438, p = 0.01208). These results suggest that OCD and hoarding may share glial-related genetic-expression architecture despite showing different directions in specific aging, complement, and pruning pathways.

GLP-1/insulin and metabolic findings

A distinct metabolic pattern emerged in OCD. Meta-analysis across brain tissues identified several gene sets meeting the permutation p < 0.05 threshold (Table 5). The strongest OCD signal was intrinsic apoptosis, followed by complement, synapse pruning, cellular senescence in the negative direction, and insulin secretion involved in the cellular response to glucose stimulus. Hoarding also showed a permutation-supported adenosine monophosphate-activated protein kinase (AMPK)-mechanistic target of rapamycin (mTOR) energy-sensing signal, consistent with the broader senescence/NAD/metabolic-aging pattern, although this should be interpreted cautiously because of pathway overlap and multiple testing.

**Table 5:** Top permutation-supported gene sets from meta-analysis across brain tissues Stouffer Z combines pathway Z-scores across six brain-region prediction models. Positive values indicate higher genetically predicted expression associated with higher trait liability; negative values indicate inverse predicted-expression association. Permutation p-values are empirical gene-label permutation values and are not equivalent to global family-wise FDR correction. OCS: Obsessive-compulsive symptoms; GOBP: Gene Ontology biological process; mTOR: Mechanistic target of rapamycin; LKB1: Liver kinase B1; AMPK: Adenosine monophosphate-activated protein kinase; FDR: False discovery rate

Disorder	Gene set	Stouffer Z	Permutation P	Direction
OCS	REACTOME_INTRINSIC_PATHWAY_FOR_APOPTOSIS	+5.707	0.004	Upregulated
OCS	GOBP_INSULIN_SECRETION (glucose response)	+4.017	0.045	Upregulated

The GLP-1-specific gene sets were not treated as a robust standalone finding because some were small and did not show the same level of statistical support as the insulin/glucose-response signal. They are retained as exploratory metabolic hypotheses rather than as a central result.

Summary of the mechanistic model

The overall pattern supports partially divergent molecular profiles. Hoarding was marked by a more positive cellular-senescence profile and portions of NAD/nicotinamide metabolism, together with AMPK-mTOR energy-sensing involvement. Obsessive-compulsive disorder, by contrast, showed a negative senescence profile, positive complement and intrinsic apoptosis signals, a negative synapse-pruning signal, and selective positive insulin secretion/glucose-response signaling. At the gene level, SIRT2, NAMPT, CAT, and MTCH2 were among the most influential candidate contributors to the difference. Mitochondrial and oxidative phosphorylation pathways were mostly neutral at the global level, whereas intrinsic apoptosis was positive in both conditions, especially in OCD. A reasonable working model is that hoarding reflects a senescence- and NAD-biased aging-related program in relevant circuits, while OCD reflects a combination of immune-pruning dysregulation, apoptotic stress, and metabolic-reward disturbance. This model remains exploratory because not all pathway findings survived correction across all possible testing families, and no colocalization or fine-mapping was performed.

## Discussion

Interpretation of key divergences

The most prominent result of this head-to-head TWAS was the opposite behavior of senescence-related pathways in the two phenotypes. Hoarding showed a more positive cellular-senescence predicted-expression profile, whereas OCD showed a negative profile in the same pathway family. This was the clearest molecular distinction in the study and fits the clinical observation that hoarding often intensifies with age, whereas OCD usually begins much earlier [2,3]. The data, therefore, support the hypothesis that genetic risk for hoarding may be more closely aligned with aging-like programs in brain circuits that govern decision-making and cognitive flexibility [10,18].

Complement and synapse pruning findings were also important, although they were somewhat more complex. Complement enrichment was positive in OCD, in line with models that implicate immune-mediated synaptic tagging in compulsive pathology, whereas the dedicated synapse pruning set moved in the opposite direction [23]. That pattern suggests that the difference between OCD and hoarding is not reducible to more or less pruning in a simple sense. Instead, different components of immune-plasticity regulation may be altered in each phenotype. This interpretation is compatible with recent proposals that OCD and hoarding engage overlapping but distinct plasticity mechanisms within cortico-striatal circuitry [28].

The NAD metabolism and SIRT biology also emerged as a point of separation. The SIRT2, NAMPT, and SIRT3 showed large opposite-direction effects, while NT5E was concordantly positive but stronger in hoarding. These observations suggest that NAD-related regulation may shape different aging and stress-response trajectories in the two conditions while still allowing some overlap in downstream metabolic effects [16,17,29]. The selective positive insulin secretion/glucose-response signal in OCD adds another layer. Unlike the stronger apoptosis, complement, and senescence findings, GLP-1-specific findings should be considered exploratory because of the small gene-set size and weaker statistical support.

Mechanistic model and circuit implications

**Figure 1:**
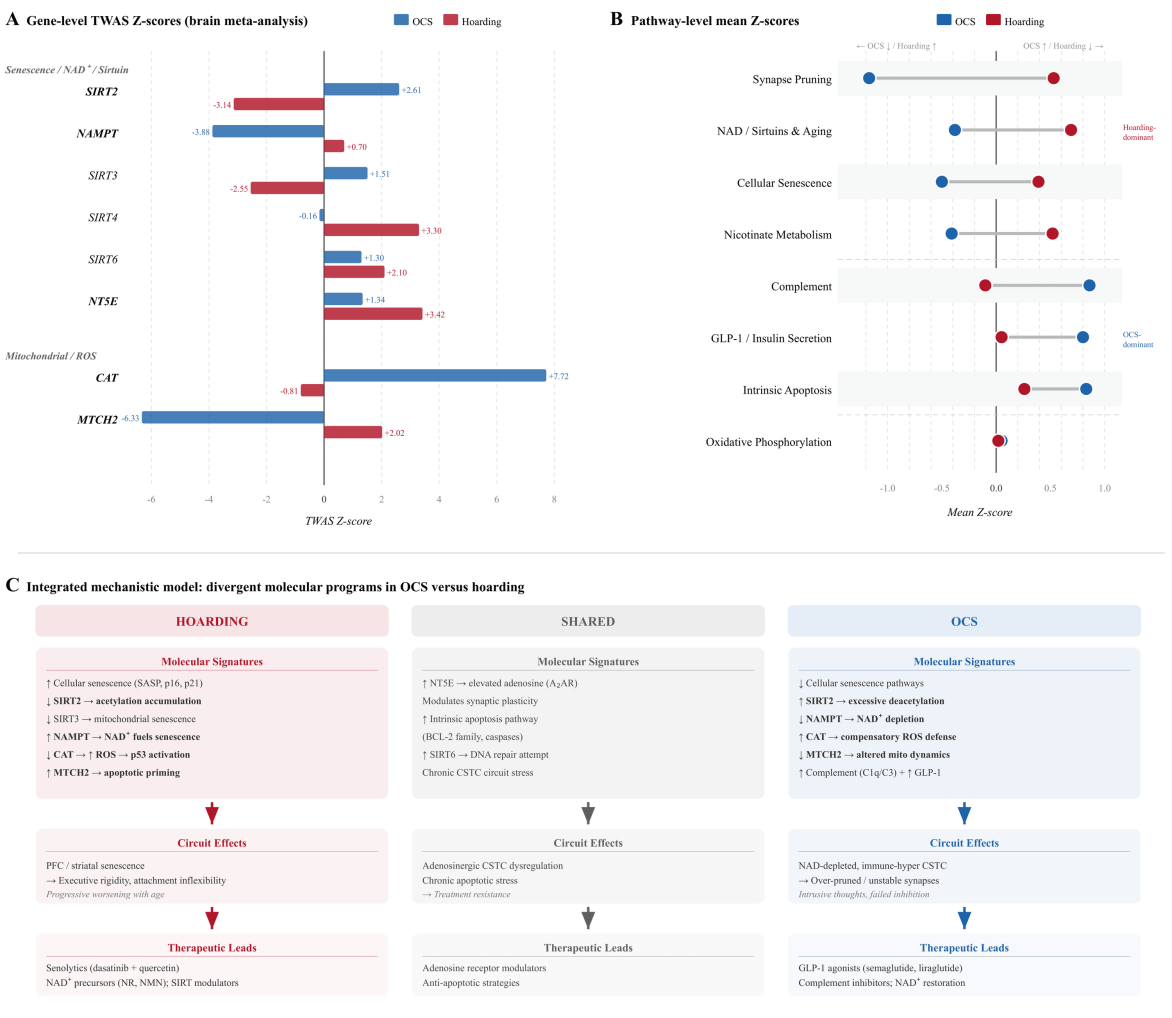
Hypothetical model of opposing molecular programs in OCD liability versus hoarding-symptom liability based on comparative TWAS A: The TWAS Z-scores for candidate genes across six brain regions, namely, the amygdala, anterior cingulate cortex, caudate, frontal cortex BA9, hippocampus, and nucleus accumbens. Positive Z-scores indicate higher genetically predicted expression associated with higher trait liability; negative scores indicate inverse predicted-expression associations. Blue bars represent OCD liability; red bars represent hoarding-symptom liability. Candidate genes include SIRT2, SIRT3, SIRT4, SIRT6, NAMPT, NT5E, CAT, and MTCH2. B: Pathway-level mean Z-scores for curated gene sets shown as a dumbbell chart, ordered by phenotype opposition. Paired OCD and hoarding dots are connected by gray lines; greater separation indicates stronger divergence. Dashed separators mark hoarding-dominant opposing pathways, OCD-dominant opposing pathways, and neutral pathways. The strongest divergence involves cellular senescence, complement, synapse pruning, and NAD/sirtuin biology. C: Integrated hypothesis shows how divergent predicted-expression signatures could map onto circuit effects and future therapeutic hypotheses. Hoarding-symptom liability is associated with a senescence- and NAD-biased aging profile in prefrontal and striatal circuits, potentially favoring executive rigidity and age-related worsening. The OCD liability is linked to complement-mediated synaptic instability, apoptotic stress, and insulin/glucose-response signaling in CSTC loops. OCD: Obsessive-compulsive disorder, NAD: Nicotinamide adenine dinucleotide, SIRT: Sirtuin, NAMPT: Nicotinamide phosphoribosyltransferase, NT5E: 5′-nucleotidase ecto, CAT: Catalase, MTCH2: , GLP-1: Glucagon-like peptide-1, SASP: Senescence-associated secretory phenotype, NR: Nicotinamide ribosid, NMN: Nicotinamide mononucleotide, PFC: Prefrontal cortex, TWAS: Transcriptome-wide association study, CSTC: Cortico-striato-thalamo-cortical Figure created by author using Microsoft PowerPoint (Microsoft Corp., Redmond, WA, USA)

At the gene level, several candidates help make the pathway findings more concrete. The SIRT2, SIRT3, SIRT4, and SIRT6 are related members of the NAD+-dependent SIRT family but differ in subcellular localization and function [16,29]. The SIRT2 is mainly cytosolic and nuclear and has been linked to microtubule and glial biology; SIRT3 and SIRT4 are mitochondrial sirtuins; and SIRT6 is mainly nuclear and involved in chromatin regulation, DNA repair, and aging-related stress responses [16,29]. The SIRT2 was predicted to be positive in OCD liability and negative in hoarding-symptom liability. Because SIRT2 has been linked to microtubule stability, tau phosphorylation, and oligodendrocyte function, lower predicted SIRT2 expression in hoarding could plausibly favor accumulation of acetylated substrates and senescence-like changes in prefrontal circuits, whereas higher SIRT2 in OCD may reflect a different pattern of synaptic or glial adaptation (Figure 1) [29].

Nicotinamide phosphoribosyltransferase is the rate-limiting enzyme in the NAD+ salvage pathway, whereas NT5E, also known as CD73, participates in extracellular nucleotide metabolism and adenosine generation. The NAMPT was strongly negative in OCD. Reduced NAMPT activity is associated with NAD+ depletion, impaired mitochondrial function, and greater neuronal vulnerability, which offers one possible explanation for why intrinsic apoptosis appeared so prominent in OCD even though senescence-related programs were genetically suppressed [17]. Other genes fit this framework as well. The CAT, which encodes catalase, was markedly positive in OCD and may reflect a compensatory antioxidant-related association. The MTCH2, a regulator of mitochondrial apoptosis priming, showed opposite-direction effects and may shift survival thresholds differently across the two conditions.

These molecular changes were most evident across caudate, nucleus accumbens, hippocampus, amygdala, anterior cingulate, and frontal cortical prediction models, all regions relevant to compulsivity models [13]. One possible interpretation is that hoarding risk promotes a slow, senescence-biased decline in flexibility and executive control, while OCD risk promotes a state marked by immune activation, apoptotic priming, and metabolic dysregulation that destabilizes inhibitory control and favors repetitive intrusive patterns. This interpretation should be treated as a hypothetical model derived from genetically predicted expression, not as direct evidence of measured cellular aging or apoptosis in patient brain tissue.

It is also important to distinguish TWAS-inferred biology from observed patient phenotypes. The current results reflect genetically predicted expression rather than measured expression in affected individuals [15]. That distinction matters because patient-level studies have reported evidence of accelerated cellular aging in OCD, including shorter telomeres and lower mitochondrial DNA copy number [14]. The present findings do not contradict those observations outright. Instead, they suggest that the inherited expression profile associated with OCD liability may differ from the downstream state seen in chronic illness, where environmental exposures, treatment history, inflammation, and disease progression may shift the biology further.

Comparison with existing literature

The pattern observed here is broadly consistent with the clinical literature. Hoarding tends to be less responsive to serotonin reuptake inhibitors and more strongly linked to age-related progression, which fits a senescence-weighted model [2,4-6,18]. Obsessive-compulsive disorder, in contrast, showed stronger complement-related and pruning-related signals, which fit neurodevelopmental and immune-plasticity models of compulsivity [11,13,23]. The current findings also align with newer plasticity frameworks that distinguish immune-mediated synaptic elimination in OCD from broader neurotrophic or metabolic enrichment in hoarding-related phenotypes [28].

Relative to broader psychiatric TWAS work, the main point here is not shared enrichment but directional contrast. Many psychiatric disorders share stress-response or mitochondrial signatures. In this comparison, the more informative result was the split between senescence- and NAD-related programs on one side and complement-, apoptosis-, pruning-, and glucose-response-related signals on the other. That degree of opposition may help explain why these conditions overlap clinically yet diverge in age course and treatment response.

Translational Implications

The findings suggest several translational possibilities, but these should be treated as future research hypotheses rather than clinical recommendations. One possibility is stratified biomarker development. Peripheral measures of NAD-related metabolites, senescence-associated secretory phenotype (SASP) factors, complement components, or incretin-related markers could eventually help identify biologically distinct subgroups across the compulsive spectrum. Imaging approaches aimed at synaptic density or senescence-associated targets might provide another route for testing these models in vivo.

For hoarding, interventions that target cellular senescence or restore NAD+ balance are especially plausible hypotheses for future study [16-18]. Senolytics are interventions intended to selectively eliminate senescent cells or reduce senescence-associated inflammatory burden. Senolytic strategies such as dasatinib plus quercetin have reached early-stage testing in Alzheimer’s disease, with evidence of feasibility and central nervous system exposure in early trials [24,25]. The NAD+ precursors, including nicotinamide riboside (NR) and nicotinamide mononucleotide (NMN), are being explored in aging and neurodegenerative settings and may be reasonable candidates for future mechanistic studies of hoarding-related biology [16,17]. The prominent role of SIRT2 in the present data also suggests that selective sirtuin modulation may deserve attention, although this remains speculative [29].

For OCD, the insulin secretion/glucose-response signature points toward metabolic-reward pathways. The GLP-1 receptor agonists such as liraglutide and semaglutide have drawn preliminary interest in conditions involving reward-related or compulsive food-related behavior, but the current data do not establish GLP-1 biology as a robust OCD treatment target [19-22]. Complement-targeting strategies may represent another future avenue, particularly in biologically enriched OCD subgroups [23]. The NAMPT-directed interventions remain largely preclinical, but they may eventually be relevant if the observed NAD+ depletion signature proves robust in follow-up studies [17].

A broader implication is that precision psychiatry in this area may require pathway-informed stratification rather than diagnosis alone. Patients with high senescence-related liability may not respond to the same interventions as patients whose biology is dominated by complement activation or apoptotic stress. That distinction could matter for trial design and for treatment development.

Strengths and limitations

A strength of this study is its direct comparative design. Rather than testing OCD and hoarding separately, the analysis contrasted the same curated gene sets across both phenotypes, allowing shared and opposing patterns to be identified. Additional strengths include the use of large GWAS summary statistics, brain-region-specific TWAS models, hypothesis-driven aging and immune pathway selection, cross-tissue meta-analysis, empirical permutation testing, and gene-level follow-up of pathway signals.

Several limitations should be kept in view. First, TWAS estimates genetically predicted expression and does not directly measure expression in patient tissue, so the results are associative rather than causal [15]. Second, the hoarding GWAS had lower power and did not yield genome-wide significant loci, which likely reduced sensitivity to weaker transcriptomic effects [7]. This power imbalance may also contribute to apparent sign flips when hoarding estimates are noisy, so opposing directions should be interpreted most cautiously for small gene sets or single-gene results.

Third, several pathway-level differences were nominal or locally corrected rather than uniformly robust after global multiple-comparison correction. Although selected differential contrasts for cellular senescence, complement, and synapse pruning were locally FDR-supported, a single family-wise correction across all pathways, tissues, cell types, and gene-level analyses was not computed. Therefore, deterministic claims about distinct disease biology would be premature.

Fourth, TWAS and pathway statistics are affected by LD, correlated eQTL weights, overlapping genes, and gene-gene dependence [15,26,27]. The Stouffer and paired-test framework used here provides a useful profile-level screen, but it does not fully account for LD-induced correlation. A TWAS association can occur when a GWAS variant and an eQTL variant are correlated through LD rather than sharing the same causal variant. Colocalization methods and TWAS fine-mapping would be needed to distinguish shared causal regulatory signals from LD-driven associations [26,27]. Fifth, the analysis relied on European-ancestry GWAS and brain eQTL reference panels, which limit generalizability. Finally, the gene-set approach was intentionally hypothesis-driven. That sharpened the test of specific mechanisms but may have left other relevant biology unexplored.

Future directions

The next step is functional and genetic validation. At the genetic level, top candidate genes and loci should be tested with colocalization, SuSiE-colocalization, eCAVIAR-like multi-signal colocalization, or FOCUS-style TWAS fine-mapping before being treated as causal genes [26,27]. Induced pluripotent stem cell-derived neurons and glia from individuals stratified by high or low pathway-specific genetic risk could be used to test whether predicted changes in SIRT2, NAMPT, CAT, or related genes produce measurable differences in senescence markers, NAD+ levels, complement-related processes, or synaptic pruning. Brain organoid or assembloid systems may be especially useful for interrogating cortico-striatal interactions. Animal studies with conditional manipulation of genes such as SIRT2, NAMPT, or CAT could help determine whether these molecular differences affect compulsive-like or hoarding-like behavior. Integrating TWAS results with proteomic, epigenomic, and longitudinal clinical data would also help distinguish upstream liability from downstream disease states.

## Conclusions

This comparative TWAS of OCD liability and hoarding-symptom liability points to partly divergent molecular profiles. Cellular senescence and NAD/sirtuin biology emerged as especially strong points of contrast, while OCD showed more prominent complement, apoptosis, synapse pruning, and insulin/glucose response signals. The most promising findings were hoarding’s more positive cellular senescence profile, OCD's intrinsic apoptosis and complement/pruning profile, and the exploratory hoarding-skewed NAD/nicotinamide signal. However, these findings should be regarded as hypothesis-generating because TWAS does not establish causality, gene-level signals were not colocalized, and global correction across all exploratory analyses was not available. More broadly, the results show the value of targeted comparative TWAS for separating related psychiatric phenotypes into more specific mechanistic models. Follow-up validation will be necessary before these findings can support biomarker or therapeutic development.

## Appendices

Appendix A

Comprehensive results of the study are presented below in Tables 6-11. 

**Table 6:** Significant gene-set enrichment findings per disease/tissue (71 findings) All gene-set names are listed without the trailing '_ALL' suffix; "pooled" denotes meta-analysis across tissues. Negative Stouffer Z values indicate down-direction effects. Stouffer Z is the meta-analytic z-score. pperm: Permutation, pW: Wilcoxon, GOBP: Gene Ontology biological process, KEGG: Kyoto Encyclopedia of Genes and Genomes, WP: WikiPathways, NAD: Nicotinamide adenine dinucleotide, AMPK: Adenosine monophosphate-activated protein kinase

#	Gene set	Disease	Tissue	n	Stouffer Z	_p__perm_	p_W_
1	GOBP_CELLULAR_SENESCENCE	hoarding	Caudate basal ganglia	50	3.141	—	0.00404
2	REACTOME_INTRINSIC_PATHWAY_FOR_APOPTOSIS	ocs	Nucleus accumbens BG	29	4.729	—	0.00479
3	REACTOME_INTRINSIC_PATHWAY_FOR_APOPTOSIS	ocs	Hippocampus	24	3.545	—	0.00533
4	GOBP_INSULIN_SECRETION_INVOLVED_IN_CELLULAR_RESPONSE_TO_GLUCOSE_STIMULUS	ocs	Caudate basal ganglia	33	4.027	—	0.00548
5	REACTOME_INTRINSIC_PATHWAY_FOR_APOPTOSIS	ocs	pooled	47	5.707	0.00590	0.04987
6	GOBP_REGULATION_OF_CELLULAR_SENESCENCE	hoarding	Hippocampus	19	2.413	—	0.00618
7	REACTOME_COMPLEMENT	ocs	Hippocampus	20	3.129	—	0.00831
8	GOBP_CELLULAR_SENESCENCE	hoarding	Amygdala	52	2.495	—	0.00942
9	GOBP_REGULATION_OF_CELLULAR_SENESCENCE	hoarding	Caudate basal ganglia	21	2.809	—	0.01135
10	REACTOME_COMPLEMENT	ocs	pooled	33	4.932	0.01640	0.01444
11	GOBP_SYNAPSE_PRUNING	ocs	pooled	17	-4.802	0.01780	0.03479
12	REACTOME_ENERGY_DEPENDENT_REGULATION_OF_MTOR_BY_LKB1_AMPK	hoarding	pooled	23	3.947	0.02430	0.1424
13	REACTOME_COMPLEMENT	ocs	Caudate basal ganglia	17	2.776	—	0.02667
14	WP_NAD_METABOLISM_SIRTUINS_AND_AGING	hoarding	Ant. cingulate cortex BA24	9	2.315	—	0.02734
15	HALLMARK_PI3K_AKT_MTOR_SIGNALING	ocs	Amygdala	46	-2.084	—	0.02792
16	GOBP_CELLULAR_SENESCENCE	ocs	pooled	75	-4.296	0.03310	0.07778
17	HALLMARK_APOPTOSIS	ocs	Nucleus accumbens BG	82	2.180	—	0.03814
18	REACTOME_NICOTINATE_METABOLISM	hoarding	Hippocampus	23	2.241	—	0.03838
19	REACTOME_ENERGY_DEPENDENT_REGULATION_OF_MTOR_BY_LKB1_AMPK	hoarding	Hippocampus	16	2.350	—	0.03864
20	WP_NAD_BIOSYNTHETIC_PATHWAYS	hoarding	Ant. cingulate cortex BA24	17	1.376	—	0.03954
21	KEGG_LTP	hoarding	Frontal cortex BA9	33	1.569	—	0.04054
22	GOBP_CELLULAR_SENESCENCE	ocs	Amygdala	50	-2.442	—	0.04398
23	GOBP_CELLULAR_SENESCENCE	ocs	Caudate basal ganglia	50	-2.068	—	0.04398
24	REACTOME_ENERGY_DEPENDENT_REGULATION_OF_MTOR_BY_LKB1_AMPK	hoarding	Ant. cingulate cortex BA24	16	2.339	—	0.04431
25	HALLMARK_PI3K_AKT_MTOR_SIGNALING	ocs	Ant. cingulate cortex BA24	55	-2.161	—	0.04434
26	KEGG_NICOTINATE_AND_NICOTINAMIDE_METABOLISM	hoarding	Nucleus accumbens BG	16	2.362	—	0.04683
27	GOBP_INSULIN_SECRETION_INVOLVED_IN_CELLULAR_RESPONSE_TO_GLUCOSE_STIMULUS	ocs	pooled	51	4.017	0.05180	0.3466
28	GOBP_SYNAPSE_PRUNING	ocs	Nucleus accumbens BG	13	-2.588	—	0.05737
29	GOBP_CELLULAR_SENESCENCE	hoarding	pooled	75	3.331	0.05769	0.03895
30	GOBP_REGULATION_OF_SYNAPTIC_PLASTICITY	hoarding	Nucleus accumbens BG	80	-2.037	—	0.05879
31	GOBP_REGULATION_OF_CELLULAR_SENESCENCE	hoarding	pooled	35	3.259	0.06499	0.03134
32	REACTOME_NICOTINATE_METABOLISM	hoarding	pooled	27	3.186	0.06929	0.1111
33	KEGG_APOPTOSIS	ocs	Hippocampus	39	2.642	—	0.07269
34	MOOTHA_MITOCHONDRIA	ocs	Amygdala	221	2.067	—	0.07549
35	GOCC_BCL_2_FAMILY_PROTEIN_COMPLEX	ocs	Nucleus accumbens BG	8	2.454	—	0.07812
36	REACTOME_COMPLEMENT	ocs	Ant. cingulate cortex BA24	18	2.841	—	0.08143
37	GOBP_CELLULAR_SENESCENCE	ocs	Ant. cingulate cortex BA24	48	-2.085	—	0.08211
38	ADULT_ASTRO	hoarding	Caudate basal ganglia	21	2.018	—	0.0822
39	REACTOME_INTRINSIC_PATHWAY_FOR_APOPTOSIS	ocs	Amygdala	24	2.140	—	0.08392
40	REACTOME_COMPLEMENT	ocs	Frontal cortex BA9	20	2.373	—	0.08969
41	GOBP_NAD_TRANSPORT	ocs	pooled	3	3.354	0.09329	—
42	REACTOME_TELOMERE	ocs	pooled	55	-3.376	0.09489	0.101
43	REACTOME_INTRINSIC_PATHWAY_FOR_APOPTOSIS	ocs	Caudate basal ganglia	34	2.467	—	0.09763
44	MONOAMINES	ocs	Amygdala	39	2.216	—	0.09855
45	ADULT_MICRO	ocs	Nucleus accumbens BG	17	-2.405	—	0.1089
46	GOBP_SYNAPSE_PRUNING	ocs	Frontal cortex BA9	13	-2.211	—	0.1272
47	MOOTHA_MITOCHONDRIA	ocs	Nucleus accumbens BG	268	2.186	—	0.1276
48	GOBP_SYNAPSE_PRUNING	ocs	Ant. cingulate cortex BA24	9	-2.287	—	0.1289
49	HALLMARK_PI3K_AKT_MTOR_SIGNALING	ocs	pooled	86	-3.006	0.1330	0.1235
50	ADULT_MICRO	ocs	pooled	21	-3.036	0.1352	0.412
51	REACTOME_INTRINSIC_PATHWAY_FOR_APOPTOSIS	ocs	Frontal cortex BA9	37	2.283	—	0.1406
52	MOOTHA_PGC	ocs	Frontal cortex BA9	222	2.054	—	0.159
53	KEGG_NICOTINATE_AND_NICOTINAMIDE_METABOLISM	hoarding	pooled	22	2.424	0.1718	0.3053
54	GOBP_CELLULAR_SENESCENCE	ocs	Frontal cortex BA9	52	-2.334	—	0.1836
55	WP_NAD_BIOSYNTHETIC_PATHWAYS	hoarding	pooled	19	2.323	0.1869	0.1688
56	WP_NAD_METABOLISM	hoarding	pooled	14	2.286	0.1917	0.2166
57	WP_NAD_METABOLISM_SIRTUINS_AND_AGING	hoarding	pooled	10	2.186	0.2138	0.1055
58	GOBP_INSULIN_SECRETION_INVOLVED_IN_CELLULAR_RESPONSE_TO_GLUCOSE_STIMULUS	ocs	Nucleus accumbens BG	36	1.970	—	0.2264
59	GOBP_SYNAPSE_PRUNING	hoarding	pooled	15	2.047	0.2372	0.1205
60	GOMF_NADPLUS_BINDING	ocs	pooled	16	2.316	0.2405	0.2979
61	GOBP_SYNAPSE_PRUNING	ocs	Caudate basal ganglia	8	-2.320	—	0.250
62	GOBP_REGULATION_OF_SYNAPTIC_PLASTICITY	hoarding	pooled	152	-2.050	0.2535	0.223
63	GOBP_REGULATION_OF_SYNAPTIC_PLASTICITY	ocs	pooled	155	2.175	0.2794	0.416
64	GOBP_INSULIN_SECRETION_INVOLVED_IN_CELLULAR_RESPONSE_TO_GLUCOSE_STIMULUS	ocs	Amygdala	31	2.404	—	0.2899
65	MOOTHA_MITOCHONDRIA	ocs	pooled	355	2.113	0.3011	0.5585
66	KEGG_APOPTOSIS	ocs	pooled	68	2.037	0.3135	0.3488
67	HALLMARK_APOPTOSIS	ocs	pooled	135	2.028	0.3139	0.1108
68	KEGG_MEDICUS_REFERENCE_EP_NE_ADRB_CAMP_SIGNALING_PATHWAY	ocs	pooled	11	2.010	0.3204	0.3652
69	GOBP_REGULATION_OF_SYNAPTIC_PLASTICITY	ocs	Amygdala	85	1.968	—	0.3243
70	GOBP_NAD_TRANSPORT	ocs	Frontal cortex BA9	3	2.019	—	—
71	GOBP_NAD_TRANSPORT	ocs	Nucleus accumbens BG	3	2.479	—	—

**Table 7:** Significant differential tests comparing OCS versus hoarding (eight pairs) MWU: Mann–Whitney U, PairedW: Paired Wilcoxon, RBC: Rank-biserial correlation, OCS: Obsessive-compulsive symptoms, GOBP: Gene Ontology biological process, KEGG: Kyoto Encyclopedia of Genes and Genomes, WP: WikiPathways, GLP1: Glucagon-like peptide-1

#	Gene set	Comparison	Mean Z diff.	MWU p	FDR	PairedW p	RBC
1	GOBP_CELLULAR_SENESCENCE	OCS vs hoarding	-0.872	0.00886	0.00886	0.04091	0.249
2	REACTOME_COMPLEMENT	OCS vs hoarding	1.059	0.0368	0.0368	0.00934	-0.305
3	GOBP_SYNAPSE_PRUNING	OCS vs hoarding	-1.176	0.03809	0.03809	0.05536	0.449
4	KEGG_NICOTINATE_AND_NICOTINAMIDE_METABOLISM	OCS vs hoarding	-0.931	0.1131	0.1131	0.03012	0.281
5	REACTOME_NICOTINATE_METABOLISM	OCS vs hoarding	-0.785	0.1828	0.1828	0.04626	0.213
6	WP_NAD_METABOLISM_SIRTUINS_AND_AGING	OCS vs hoarding	-1.066	0.1859	0.1859	0.2754	0.360
7	GOBP_NAD_TRANSPORT	OCS vs hoarding	2.375	0.700	0.700	0.250	-0.333
8	WP_GLP1_FROM_INTESTINE_AND_PANCREAS_AND_ROLE_IN_GLUCOSE_HOMEOSTASIS	OCS vs hoarding	1.562	0.700	0.700	0.750	-0.333

**Table 8:** Significant proximity pairs between OCS and hoarding gene-set profiles (six pairs) OCS: Obsessive-compulsive symptoms, cos: Cosine similarity, GOBP: Gene Ontology biological process, NAD: Nicotinamide adenine dinucleotide

#	Gene set	Comparison	n	Pearson r	p_r_	Spearman ρ	p_ρ_	cos
1	ADULT_MICRO	OCS vs hoarding	20	0.636	0.00258	0.603	0.00489	0.609
2	WP_GLP1_FROM_INTESTINE_AND_PANCREAS_AND_ROLE_IN_GLUCOSE_HOMEOSTASIS	OCS vs hoarding	3	-1.000	0.02006	-1.000	0	-0.998
3	ADULT_ASTRO	OCS vs hoarding	32	0.373	0.03551	0.438	0.01208	0.377
4	REACTOME_SIRT1_NEGATIVELY_REGULATES_RRNA_EXPRESSION	OCS vs hoarding	7	0.765	0.04514	0.464	0.2939	0.665
5	WP_NAD_BIOSYNTHETIC_PATHWAYS	OCS vs hoarding	19	-0.449	0.0536	-0.481	0.03722	-0.439
6	GOBP_NAD_TRANSPORT	OCS vs hoarding	3	0.965	0.1703	1.000	0	0.699

**Table 9:** Significant pairwise comparisons between OCS and hoarding (14 pairs) Key supporting p-values are listed; dp: Paired Cohen's d; du: Unpaired Cohen's d, OCS: Obsessive-compulsive symptoms, GOBP: Gene Ontology biological process, KEGG: Kyoto Encyclopedia of Genes and Genomes, WP: WikiPathways, NAD: Nicotinamide adenine dinucleotide,

#	Gene set	n_p_	Mean diff.	d_p_	d_u_	Key p-values / notes
1	MOOTHA_MITOCHONDRIA	352	0.123	0.04	0.06	Levene p = 0.000386 (variance shift)
2	REACTOME_COMPLEMENT	32	1.059	0.51	0.49	MWU 0.0335; Welch t 0.0489; Brunner-Munzel 0.0289; paired t 0.00673; PairedW 0.00934; perm 0.00510
3	GOBP_CELLULAR_SENESCENCE	74	-0.872	-0.29	-0.42	MWU 0.00665; KS 0.0263; Welch t 0.0114; Brunner-Munzel 0.00559; paired t 0.0153; PairedW 0.0409; perm 0.0144
4	GOBP_SYNAPSE_PRUNING	15	-1.176	-0.56	-1.00	MWU 0.0141; KS 0.0177; Welch t 0.00826; Brunner-Munzel 0.00820; paired t 0.0478; perm 0.0486
5	KEGG_LTP	52	0.003	0.00	0.00	Levene p = 0.0121 (variance shift)
6	KEGG_NICOTINATE_AND_NICOTINAMIDE_METABOLISM	22	-0.931	-0.53	-0.60	paired t 0.0208; PairedW 0.0301; perm 0.0228
7	GOBP_INTRINSIC_APOPTOTIC_SIGNALING_PATHWAY	260	0.073	0.03	0.03	Levene p = 0.0210 (variance shift)
8	REACTOME_INTRINSIC_PATHWAY_FOR_APOPTOSIS	47	0.574	0.21	0.28	Levene p = 0.0321 (variance shift)
9	KEGG_APOPTOSIS	67	0.036	0.01	0.01	Levene p = 0.0333 (variance shift)
10	REACTOME_NICOTINATE_METABOLISM	27	-0.785	-0.39	-0.46	PairedW 0.0463
11	GOBP_GLUTAMATERGIC	73	0.100	0.04	0.06	Levene p = 0.0493 (variance shift)
12	GOBP_NAD_TRANSPORT	3	2.375	1.22	0.72	large effect; small n
13	WP_NAD_METABOLISM_SIRTUINS_AND_AGING	10	-1.066	-0.39	-0.65	large unpaired effect
14	REACTOME_SIRT1_NEGATIVELY_REGULATES_RRNA_EXPRESSION	7	-0.838	-0.61	-0.44	large paired effect

**Table 10:** Significant concordance pairs between OCS and hoarding (five pairs) CCC: Lin's concordance correlation coefficient, GOBP: Gene Ontology biological process, WP: WikiPathways, NAD: Nicotinamide adenine dinucleotide, AMPK: Adenosine monophosphate-activated protein kinase

#	Gene set	n_p_	Sign rate	_p__sign_	CCC	Kendall τ	p_τ_
1	ADULT_MICRO	20	0.684	0.1671	0.599	0.463	0.00378
2	WP_GLP1_FROM_INTESTINE_AND_PANCREAS_AND_ROLE_IN_GLUCOSE_HOMEOSTASIS	3	0.000	—	-0.767	-1.000	0.3333
3	REACTOME_SIRT1_NEGATIVELY_REGULATES_RRNA_EXPRESSION	7	0.571	1	0.677	0.429	0.2389
4	GOBP_CAMKK_AMPK_SIGNALING_CASCADE	8	0.250	0.2891	-0.550	-0.357	0.2751
5	GOBP_NAD_TRANSPORT	3	0.667	1	0.660	1.000	0.3333

**Table 11:** Genes whose leave-one-out removal flips the sign of the meta-analytic Stouffer Z (i.e., the gene's contribution dominates direction). Of the 1,791 influential gene–disease entries, only the SIGN-FLIP subset is shown. ΔStouffer is the change in Stouffer Z when the gene is removed; the percentage in parentheses is the relative effect on the overall statistic. ΔStouffer is the change in Stouffer Z when the gene is removed; the percentage in parentheses is the relative effect on the overall statistic. Sign-flip indicates that removing the gene reverses the direction (sign) of the meta-analytic Stouffer Z. Genes are tagged across all flagged gene sets in the source file (ADULT_*, GOBP_*, GOMF_*, HALLMARK_*, KEGG_*, MOOTHA_*, REACTOME_*, SAUL_SEN_MAYO, WANG_*, WP_*). Long gene-set names are abbreviated with ellipses for layout. Δ% denotes percentage change in the overall Stouffer statistic upon LOO removal. GOBP: Gene Ontology biological process, OCS: Obsessive-compulsive symptoms, WP: WikiPathways, NAD: Nicotinamide adenine dinucleotide, AMPK: Adenosine monophosphate-activated protein kinase, GLP1: Glucagon-like peptide-1

#	Gene set	Disease	Gene	Z	ΔStouffer	Δ%
1	ADULT_MICRO	hoarding	A2M	3.65	0.83	233.5
2	ADULT_MICRO	hoarding	ITGAX	2.58	0.58	164.7
3	ADULT_MICRO	hoarding	LAPTM5	1.68	0.38	106.4
4	ADULT_OLIGO	hoarding	GLYCTK	3.58	0.76	584.7
5	ADULT_OLIGO	hoarding	PLEKHB1	1.78	0.38	288.7
6	ADULT_OLIGO	hoarding	MSX1	1.68	0.36	272.8
7	ADULT_OLIGO	hoarding	GPNMB	0.98	0.21	158.8
8	ADULT_OLIGO	hoarding	MBP	0.88	0.19	142.5
9	ADULT_OLIGO	hoarding	ABCA8	0.73	0.15	117.4
10	ADULT_OLIGO	hoarding	KCNMB4	0.72	0.15	115.4
11	GOBP_CAMKK_AMPK_SIGNALING_CASCADE	hoarding	LRRK2	2.94	1.05	128.4
12	GOBP_CAMKK_AMPK_SIGNALING_CASCADE	OCS	TREM2	-4.35	-1.41	170.6
13	GOBP_CAMKK_AMPK_SIGNALING_CASCADE	OCS	LRRK2	-3.01	-0.96	116.2
14	GOBP_GLUTAMATERGIC	OCS	RAB3GAP1	6.75	0.78	562.2
15	GOBP_GLUTAMATERGIC	OCS	NTRK1	3.97	0.46	330.5
16	GOBP_GLUTAMATERGIC	OCS	GRID1	3.18	0.37	264.3
17	GOBP_GLUTAMATERGIC	OCS	STXBP1	3.16	0.37	262.8
18	GOBP_GLUTAMATERGIC	OCS	NLGN2	2.92	0.34	243.2
19	GOBP_GLUTAMATERGIC	OCS	ABCC8	2.89	0.33	240.1
20	GOBP_GLUTAMATERGIC	OCS	SLC17A6	2.86	0.33	237.9
21	GOBP_GLUTAMATERGIC	OCS	GRM3	2.82	0.33	234.8
22	GOBP_GLUTAMATERGIC	OCS	SYT1	2.04	0.24	169.3
23	GOBP_GLUTAMATERGIC	OCS	CLN3	2.01	0.23	167.0
24	GOBP_GLUTAMATERGIC	OCS	GRIA4	1.99	0.23	165.6
25	GOBP_GLUTAMATERGIC	OCS	ADORA1	1.99	0.23	165.2
26	GOBP_GLUTAMATERGIC	OCS	KMO	1.73	0.20	143.9
27	GOBP_GLUTAMATERGIC	OCS	PNOC	1.63	0.19	135.5
28	GOBP_GLUTAMATERGIC	OCS	ATP1A2	1.60	0.18	132.7
29	GOBP_GLUTAMATERGIC	OCS	DRD2	1.58	0.18	131.5
30	GOBP_GLUTAMATERGIC	OCS	SLC17A8	1.37	0.16	113.7
31	GOBP_GLUTAMATERGIC	OCS	PSEN1	1.27	0.15	105.7
32	GOBP_GLUTAMATERGIC	OCS	GRIN1	1.26	0.15	104.3
33	GOBP_GLUTAMATERGIC	OCS	DSCAM	1.24	0.14	102.8
34	GOBP_INSULIN_SECRETION_…GLUCOSE	hoarding	PTPRN	4.37	0.59	289.0
35	GOBP_INSULIN_SECRETION_…GLUCOSE	hoarding	SIDT2	3.26	0.44	215.4
36	GOBP_INSULIN_SECRETION_…GLUCOSE	hoarding	STX4	2.76	0.37	181.9
37	GOBP_INSULIN_SECRETION_…GLUCOSE	hoarding	RAB11FIP5	2.70	0.37	178.1
38	GOBP_INSULIN_SECRETION_…GLUCOSE	hoarding	PPARD	2.65	0.36	175.1
39	GOBP_INSULIN_SECRETION_…GLUCOSE	hoarding	PIM3	2.05	0.28	134.8
40	GOBP_INSULIN_SECRETION_…GLUCOSE	hoarding	BAIAP3	1.90	0.26	125.4
41	GOBP_INSULIN_SECRETION_…GLUCOSE	hoarding	ALDOA	1.90	0.26	125.0
42	GOBP_INSULIN_SECRETION_…GLUCOSE	hoarding	BAD	1.86	0.25	122.2
43	GOBP_INSULIN_SECRETION_…GLUCOSE	hoarding	NR1D1	1.83	0.25	120.2
44	GOBP_INSULIN_SECRETION_…GLUCOSE	hoarding	PTPRN2	1.65	0.22	108.4
45	GOBP_INSULIN_SECRETION_…GLUCOSE	hoarding	HIF1A	1.56	0.21	102.6
46	GOBP_INTRINSIC_APOPTOTIC_SIGNALING	OCS	MTCH2	-6.33	-0.39	524.1
47	GOBP_INTRINSIC_APOPTOTIC_SIGNALING	OCS	MAP2K1	-5.65	-0.35	467.8
48	GOBP_INTRINSIC_APOPTOTIC_SIGNALING	OCS	FNIP2	-5.53	-0.34	458.0
49	GOBP_INTRINSIC_APOPTOTIC_SIGNALING	OCS	TRIM32	-5.42	-0.33	449.2
50	GOBP_INTRINSIC_APOPTOTIC_SIGNALING	OCS	OPA1	-4.93	-0.30	408.6
51	GOBP_INTRINSIC_APOPTOTIC_SIGNALING	OCS	HDAC1	-4.43	-0.27	367.0
52	GOBP_INTRINSIC_APOPTOTIC_SIGNALING	OCS	CD74	-4.38	-0.27	362.8
53	GOBP_INTRINSIC_APOPTOTIC_SIGNALING	OCS	TREM2	-4.35	-0.27	360.8
54	GOBP_INTRINSIC_APOPTOTIC_SIGNALING	OCS	PYCARD	-4.16	-0.26	344.2
55	GOBP_INTRINSIC_APOPTOTIC_SIGNALING	OCS	HRAS	-4.03	-0.25	333.5
56	GOBP_INTRINSIC_APOPTOTIC_SIGNALING	OCS	IVNS1ABP	-3.92	-0.24	324.8
57	GOBP_INTRINSIC_APOPTOTIC_SIGNALING	OCS	MSH6	-3.63	-0.22	300.6
58	GOBP_INTRINSIC_APOPTOTIC_SIGNALING	OCS	NBN	-3.49	-0.22	289.3
59	GOBP_INTRINSIC_APOPTOTIC_SIGNALING	OCS	HNRNPK	-3.38	-0.21	280.0
60	GOBP_INTRINSIC_APOPTOTIC_SIGNALING	OCS	IFI27L1	-3.23	-0.20	267.6
61	GOBP_INTRINSIC_APOPTOTIC_SIGNALING	OCS	PIK3R1	-3.09	-0.19	256.0
62	GOBP_INTRINSIC_APOPTOTIC_SIGNALING	OCS	LRRK2	-3.01	-0.19	249.1
63	GOBP_INTRINSIC_APOPTOTIC_SIGNALING	OCS	PMAIP1	-2.88	-0.18	238.7
64	GOBP_INTRINSIC_APOPTOTIC_SIGNALING	OCS	ERCC6	-2.88	-0.18	238.4
65	GOBP_INTRINSIC_APOPTOTIC_SIGNALING	OCS	TRIB3	-2.85	-0.18	236.4
66	GOBP_INTRINSIC_APOPTOTIC_SIGNALING	OCS	PPP1R15A	-2.84	-0.17	235.1
67	GOBP_INTRINSIC_APOPTOTIC_SIGNALING	OCS	ARMC10	-2.78	-0.17	230.2
68	GOBP_INTRINSIC_APOPTOTIC_SIGNALING	OCS	PARK7	-2.69	-0.17	222.7
69	GOBP_INTRINSIC_APOPTOTIC_SIGNALING	OCS	GRINA	-2.66	-0.16	219.9
70	GOBP_INTRINSIC_APOPTOTIC_SIGNALING	OCS	CEBPB	-2.58	-0.16	213.9
71	GOBP_INTRINSIC_APOPTOTIC_SIGNALING	OCS	WWOX	-2.52	-0.16	208.8
72	GOBP_INTRINSIC_APOPTOTIC_SIGNALING	OCS	SFPQ	-2.49	-0.15	206.2
73	GOBP_INTRINSIC_APOPTOTIC_SIGNALING	OCS	SELENOS	-2.46	-0.15	203.7
74	GOBP_INTRINSIC_APOPTOTIC_SIGNALING	OCS	RTKN2	-2.38	-0.15	197.2
75	GOBP_INTRINSIC_APOPTOTIC_SIGNALING	OCS	TNFRSF1B	-2.37	-0.15	196.5
76	GOBP_INTRINSIC_APOPTOTIC_SIGNALING	OCS	IKBKE	-2.26	-0.14	186.8
77	GOBP_INTRINSIC_APOPTOTIC_SIGNALING	OCS	ING2	-2.16	-0.13	179.2
78	GOBP_INTRINSIC_APOPTOTIC_SIGNALING	OCS	CUL1	-2.16	-0.13	178.6
79	GOBP_INTRINSIC_APOPTOTIC_SIGNALING	OCS	PARP1	-2.11	-0.13	174.7
80	GOBP_INTRINSIC_APOPTOTIC_SIGNALING	OCS	BAK1	-2.09	-0.13	172.9
81	GOBP_INTRINSIC_APOPTOTIC_SIGNALING	OCS	S100A8	-2.07	-0.13	171.6
82	GOBP_INTRINSIC_APOPTOTIC_SIGNALING	OCS	NUPR1	-2.02	-0.12	167.3
83	GOBP_INTRINSIC_APOPTOTIC_SIGNALING	OCS	BID	-1.96	-0.12	162.4
84	GOBP_INTRINSIC_APOPTOTIC_SIGNALING	OCS	TP53	-1.93	-0.12	160.0
85	GOBP_INTRINSIC_APOPTOTIC_SIGNALING	OCS	CUL2	-1.91	-0.12	158.1
86	GOBP_INTRINSIC_APOPTOTIC_SIGNALING	OCS	EI24	-1.77	-0.11	146.8
87	GOBP_INTRINSIC_APOPTOTIC_SIGNALING	OCS	FBXW7	-1.76	-0.11	145.8
88	GOBP_INTRINSIC_APOPTOTIC_SIGNALING	OCS	HINT1	-1.70	-0.10	141.1
89	GOBP_INTRINSIC_APOPTOTIC_SIGNALING	OCS	TNFRSF10B	-1.70	-0.10	140.8
90	GOBP_INTRINSIC_APOPTOTIC_SIGNALING	OCS	JMY	-1.67	-0.10	138.2
91	GOBP_INTRINSIC_APOPTOTIC_SIGNALING	OCS	SOD2	-1.66	-0.10	137.8
92	GOBP_INTRINSIC_APOPTOTIC_SIGNALING	OCS	RRP8	-1.62	-0.10	134.1
93	GOBP_INTRINSIC_APOPTOTIC_SIGNALING	OCS	CDKN2D	-1.60	-0.10	132.6
94	GOBP_INTRINSIC_APOPTOTIC_SIGNALING	OCS	POU4F1	-1.53	-0.09	126.7
95	GOBP_INTRINSIC_APOPTOTIC_SIGNALING	OCS	STYXL1	-1.52	-0.09	125.8
96	GOBP_INTRINSIC_APOPTOTIC_SIGNALING	OCS	WNT1	-1.49	-0.09	123.5
97	GOBP_INTRINSIC_APOPTOTIC_SIGNALING	OCS	EIF2AK3	-1.47	-0.09	121.8
98	GOBP_INTRINSIC_APOPTOTIC_SIGNALING	OCS	HELLS	-1.45	-0.09	119.7
99	GOBP_INTRINSIC_APOPTOTIC_SIGNALING	OCS	PERP	-1.44	-0.09	119.0
100	GOBP_INTRINSIC_APOPTOTIC_SIGNALING	OCS	CTNNB1	-1.35	-0.08	111.7
101	GOBP_INTRINSIC_APOPTOTIC_SIGNALING	OCS	DDX5	-1.32	-0.08	109.1
102	GOBP_INTRINSIC_APOPTOTIC_SIGNALING	OCS	NFATC4	-1.31	-0.08	108.6
103	GOBP_INTRINSIC_APOPTOTIC_SIGNALING	OCS	SELENOK	-1.29	-0.08	106.8
104	GOBP_INTRINSIC_APOPTOTIC_SIGNALING	OCS	CCAR2	-1.26	-0.08	104.5
105	GOBP_INTRINSIC_APOPTOTIC_SIGNALING	OCS	BDKRB2	-1.21	-0.07	100.2
106	GOBP_REGULATION_OF_CELLULAR_SENESCENCE	OCS	YPEL3	-8.46	-1.47	601.4
107	GOBP_REGULATION_OF_CELLULAR_SENESCENCE	OCS	TERF2	-5.07	-0.88	359.9
108	GOBP_REGULATION_OF_CELLULAR_SENESCENCE	OCS	PAWR	-3.99	-0.69	283.1
109	GOBP_REGULATION_OF_CELLULAR_SENESCENCE	OCS	EEF1E1	-2.75	-0.48	194.5
110	GOBP_REGULATION_OF_CELLULAR_SENESCENCE	OCS	ING2	-2.16	-0.37	152.7
111	GOBP_REGULATION_OF_CELLULAR_SENESCENCE	OCS	TP53	-1.93	-0.33	136.2
112	GOBP_REGULATION_OF_CELLULAR_SENESCENCE	OCS	WNT1	-1.49	-0.26	104.8
113	GOCC_BCL_2_FAMILY_PROTEIN_COMPLEX	OCS	BAD	5.44	1.74	128.6
114	GOMF_NADPLUS_BINDING	hoarding	HADH	4.61	1.16	124.2
115	GOMF_NADPLUS_BINDING	hoarding	PARP14	4.13	1.03	110.9
116	HALLMARK_APOPTOSIS	hoarding	CASP7	4.24	0.37	140.7
117	HALLMARK_APOPTOSIS	hoarding	HMGB2	3.96	0.35	131.4
118	HALLMARK_APOPTOSIS	hoarding	LGALS3	3.80	0.33	126.1
119	HALLMARK_APOPTOSIS	hoarding	ROCK1	3.34	0.29	110.8
120	HALLMARK_APOPTOSIS	hoarding	GUCY2D	3.07	0.27	101.8
121	HALLMARK_OXIDATIVE_PHOSPHORYLATION	OCS	NDUFA2	7.53	0.61	106.9
122	KEGG_LTP	OCS	PPP1CB	6.12	0.84	137.9
123	KEGG_LTP	OCS	MAPK3	6.06	0.84	136.7
124	KEGG_MEDICUS_…ADRB_CAMP	hoarding	PRKACA	4.20	1.22	133.0
125	MOOTHA_MITOCHONDRIA	hoarding	MRPS12	-6.24	-0.33	185.4
126	MOOTHA_MITOCHONDRIA	hoarding	MIPEP	-6.07	-0.32	180.4
127	MOOTHA_MITOCHONDRIA	hoarding	ME3	-4.99	-0.26	148.1
128	MOOTHA_MITOCHONDRIA	hoarding	ETFDH	-4.69	-0.25	139.2
129	MOOTHA_MITOCHONDRIA	hoarding	CNNM4	-4.10	-0.22	121.6
130	MOOTHA_MITOCHONDRIA	hoarding	GPX4	-4.06	-0.22	120.6
131	MOOTHA_MITOCHONDRIA	hoarding	MRPL4	-3.80	-0.20	112.7
132	MOOTHA_MITOCHONDRIA	hoarding	CRAT	-3.72	-0.20	110.5
133	MOOTHA_MITOCHONDRIA	hoarding	FIBP	-3.53	-0.19	104.9
134	MOOTHA_MITOCHONDRIA	hoarding	TIMM10	-3.50	-0.19	103.8
135	MOOTHA_MITOCHONDRIA	hoarding	COX5A	-3.45	-0.18	102.5
136	REACTOME_ENERGY_DEPENDENT_REGULATION_OF_MTOR_BY_LKB1_AMPK	OCS	LAMTOR3	6.89	1.43	243.1
137	REACTOME_ENERGY_DEPENDENT_REGULATION_OF_MTOR_BY_LKB1_AMPK	OCS	PRKAB1	3.91	0.80	136.9
138	REACTOME_REGULATION_OF_INSULIN_SECRETION	hoarding	PRKACA	4.20	0.56	726.2
139	REACTOME_REGULATION_OF_INSULIN_SECRETION	hoarding	GNG10	3.89	0.51	672.9
140	REACTOME_REGULATION_OF_INSULIN_SECRETION	hoarding	PRKAR2A	3.71	0.49	641.5
141	REACTOME_REGULATION_OF_INSULIN_SECRETION	hoarding	PRKAR1A	2.15	0.28	372.4
142	REACTOME_REGULATION_OF_INSULIN_SECRETION	hoarding	CACNA1A	2.14	0.28	370.1
143	REACTOME_REGULATION_OF_INSULIN_SECRETION	hoarding	GNB5	2.12	0.28	365.9
144	REACTOME_REGULATION_OF_INSULIN_SECRETION	hoarding	STXBP1	1.96	0.26	339.2
145	REACTOME_REGULATION_OF_INSULIN_SECRETION	hoarding	ITPR3	1.96	0.26	338.4
146	REACTOME_REGULATION_OF_INSULIN_SECRETION	hoarding	GNB4	1.94	0.26	334.7
147	REACTOME_REGULATION_OF_INSULIN_SECRETION	hoarding	GNGT2	1.85	0.24	319.6
148	REACTOME_REGULATION_OF_INSULIN_SECRETION	hoarding	SNAP25	1.82	0.24	315.1
149	REACTOME_REGULATION_OF_INSULIN_SECRETION	hoarding	STX1A	1.58	0.21	272.4
150	REACTOME_REGULATION_OF_INSULIN_SECRETION	hoarding	AHCYL1	1.36	0.18	235.1
151	REACTOME_REGULATION_OF_INSULIN_SECRETION	hoarding	GNG2	1.19	0.16	205.8
152	REACTOME_REGULATION_OF_INSULIN_SECRETION	hoarding	KCNG2	1.17	0.15	202.2
153	REACTOME_REGULATION_OF_INSULIN_SECRETION	hoarding	CACNA1E	1.11	0.15	191.7
154	REACTOME_REGULATION_OF_INSULIN_SECRETION	hoarding	PLCB3	0.93	0.12	160.9
155	REACTOME_REGULATION_OF_INSULIN_SECRETION	hoarding	GNG8	0.72	0.09	123.5
156	REACTOME_REGULATION_OF_INSULIN_SECRETION	hoarding	GNG4	0.70	0.09	120.2
157	REACTOME_REGULATION_OF_INSULIN_SECRETION	hoarding	ADCY6	0.65	0.09	111.9
158	REACTOME_REGULATION_OF_INSULIN_SECRETION	hoarding	CACNB3	0.64	0.08	110.5
159	REACTOME_REGULATION_OF_INSULIN_SECRETION	hoarding	GNAQ	0.64	0.08	109.3
160	REACTOME_REGULATION_OF_INSULIN_SECRETION	hoarding	CACNA1C	0.61	0.08	104.5
161	REACTOME_SIRT1_NEGATIVELY_REGULATES_RRNA_EXPRESSION	OCS	TAF1C	-3.72	-1.48	301.2
162	REACTOME_SIRT1_NEGATIVELY_REGULATES_RRNA_EXPRESSION	OCS	RRP8	-1.62	-0.62	126.5
163	REACTOME_SYNTHESIS_…GLP1	hoarding	GNB3	-2.94	-0.87	258.1
164	REACTOME_SYNTHESIS_…GLP1	hoarding	FFAR4	-1.46	-0.42	125.6
165	REACTOME_SYNTHESIS_…GLP1	hoarding	FFAR1	-1.36	-0.40	117.3
166	REACTOME_SYNTHESIS_…GLP1	hoarding	SEC11A	-1.25	-0.36	107.5
167	REACTOME_SYNTHESIS_…GLP1	OCS	PAX6	4.72	1.41	361.5
168	REACTOME_SYNTHESIS_…GLP1	OCS	FFAR1	2.68	0.79	203.1
169	REACTOME_SYNTHESIS_…GLP1	OCS	FFAR4	2.46	0.73	186.5
170	REACTOME_SYNTHESIS_…GLP1	OCS	DPP4	1.45	0.42	107.7
171	REACTOME_SYNTHESIS_…GLP1	OCS	SEC11C	1.35	0.39	100.2
172	REACTOME_TELOMERE	hoarding	POLD3	4.27	0.58	115.2
173	REACTOME_TELOMERE	hoarding	PPP6C	4.24	0.57	114.4
174	REACTOME_TELOMERE	hoarding	SHQ1	4.00	0.54	108.0
175	SAUL_SEN_MAYO	OCS	SPX	4.06	0.43	546.7
176	SAUL_SEN_MAYO	OCS	TNFRSF1A	3.72	0.39	501.7
177	SAUL_SEN_MAYO	OCS	MMP9	3.63	0.38	489.4
178	SAUL_SEN_MAYO	OCS	SEMA3F	2.91	0.31	391.8
179	SAUL_SEN_MAYO	OCS	FAS	2.66	0.28	358.1
180	SAUL_SEN_MAYO	OCS	EGF	2.50	0.26	336.9
181	SAUL_SEN_MAYO	OCS	PTGES	2.47	0.26	332.8
182	SAUL_SEN_MAYO	OCS	PLAUR	2.26	0.24	304.8
183	SAUL_SEN_MAYO	OCS	EDN1	2.24	0.24	301.5
184	SAUL_SEN_MAYO	OCS	EGFR	2.12	0.22	285.0
185	SAUL_SEN_MAYO	OCS	NRG1	2.08	0.22	280.3
186	SAUL_SEN_MAYO	OCS	IGFBP4	2.06	0.22	277.8
187	SAUL_SEN_MAYO	OCS	IL13	1.87	0.20	252.1
188	SAUL_SEN_MAYO	OCS	IL1A	1.81	0.19	243.2
189	SAUL_SEN_MAYO	OCS	TUBGCP2	1.79	0.19	241.4
190	SAUL_SEN_MAYO	OCS	MIF	1.69	0.18	227.5
191	SAUL_SEN_MAYO	OCS	SCAMP4	1.68	0.18	226.0
192	SAUL_SEN_MAYO	OCS	FGF1	1.42	0.15	191.0
193	SAUL_SEN_MAYO	OCS	IL6	1.31	0.14	175.8
194	SAUL_SEN_MAYO	OCS	CXCL1	1.24	0.13	166.6
195	SAUL_SEN_MAYO	OCS	CXCL2	1.17	0.12	157.8
196	SAUL_SEN_MAYO	OCS	IGFBP7	1.10	0.12	147.6
197	SAUL_SEN_MAYO	OCS	MMP2	1.01	0.11	135.5
198	SAUL_SEN_MAYO	OCS	ANGPTL4	0.97	0.10	131.0
199	SAUL_SEN_MAYO	OCS	CSF2RB	0.96	0.10	128.7
200	SAUL_SEN_MAYO	OCS	CCL20	0.79	0.08	106.7
201	SAUL_SEN_MAYO	OCS	SPP1	0.79	0.08	106.1
202	WANG_ADIPOGENIC_GENES_REPRESSED_BY_SIRT1	hoarding	IGFBP4	-4.23	-0.98	160.8
203	WANG_ADIPOGENIC_GENES_REPRESSED_BY_SIRT1	hoarding	TRIB3	-3.22	-0.74	121.8
204	WP_GLP1_…GLUCOSE_HOMEOSTASIS	OCS	PCSK2	2.71	1.52	479.1
205	WP_NAD_BIOSYNTHETIC_PATHWAYS	OCS	NAMPT	-3.88	-0.91	392.3
206	WP_NAD_BIOSYNTHETIC_PATHWAYS	OCS	PARP2	-3.11	-0.73	313.3
207	WP_NAD_BIOSYNTHETIC_PATHWAYS	OCS	NAPRT	-2.21	-0.52	222.3
208	WP_NAD_BIOSYNTHETIC_PATHWAYS	OCS	PARP1	-2.11	-0.49	211.9
209	WP_NAD_BIOSYNTHETIC_PATHWAYS	OCS	BST1	-1.26	-0.29	126.0
210	WP_NAD_METABOLISM	OCS	SIRT2	2.61	0.70	106.4
211	WP_NAD_METABOLISM	OCS	NMNAT2	2.47	0.66	100.7
212	WP_NAD_METABOLISM_SIRTUINS_AND_AGING	OCS	NAMPT	-3.88	-1.23	103.7
